# Global Diversification Rates of Ferns Across Spatial and Climatic Gradients

**DOI:** 10.1002/advs.202508106

**Published:** 2025-11-05

**Authors:** Hong Qian, Michael Kessler, Shenhua Qian

**Affiliations:** ^1^ Research and Collections Center Illinois State Museum 1011 East Ash Street Springfield IL 62703 USA; ^2^ Department of Systematic and Evolutionary Botany University of Zurich Zurich 8008 Switzerland; ^3^ Key Laboratory of the Three Gorges Reservoir Region's Eco‐Environment Ministry of Education Chongqing University Chongqing 400045 China; ^4^ College of Environment and Ecology Chongqing University Chongqing 400045 China

**Keywords:** biome, latitudinal diversity gradient, pteridophyte, speciation, species richness

## Abstract

Geographic patterns of diversity in any group of plants are the result of the interplay of environmental conditions and the evolutionary dynamics of the respective plant group. Here, the geographic distribution of current mean diversification rates (MDR) is explored at the genus level for ferns and relate it to climatic conditions and regional species richness. It is found that MDR is highest at tropical latitudes and in humid and hot environments, and is influenced primarily by current climate (rather than historical climate change), by precipitation‐related variables (rather than temperature‐related ones), and roughly equally by climate extremes and seasonality. Furthermore, a positive relationship between MDR and fern species density is found, with the latter being more strongly directly influenced by MDR than by climate, and all of the above‐mentioned patterns differ among longitudinal segments. Critically, the relationship between MDR and climate shifts across longitude, revealing region‐specific diversification drivers. This study shows that diversification rates provide complementary information on the evolutionary history of ferns compared to species richness and phylogenetic diversity, and that highly diverse regional fern assemblages appear to be centers of ferns belonging to rapidly diversifying lineages.

## Graphical abstract

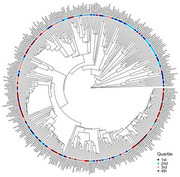

## 1 Introduction

Global patterns of animal and plant diversity are the result of the combined effects of the availability of habitats and resources, which set limits to the carrying capacity of an ecosystem, and evolutionary dynamics, which provide the species inhabiting these ecosystems.^[^ ^1^, ^2^, ^3^ ^]^ The latter involve numerous processes, from the origination of major evolutionary lineages and their adaptation to novel environments to effects of extinction and current speciation events.^[^ ^4^ ^]^ These interactions and their temporal and spatial dynamics have been extensively studied for several groups of animals (e.g.,^[^ ^5^, ^6^ ^]^) and for angiosperms (e.g.,^[^ ^7^, ^8^ ^]^).

Whereas the early evolutionary diversification dynamics, e.g., those during the origin of angiosperms, have received attention for decades, tip diversification rates, i.e., the current rates at which species are evolving, have only been explored more recently. In birds, for example, diversification rates increase with elevation in mountains where species richness is low, suggesting that these habitats have empty niche space available for speciation.^[^ ^9^ ^]^ In contrast, among liverworts, diversification rates are highest at low elevations, where a liverwort lineage is undergoing an active adaptive radiation in the rainforest canopy.^[^ ^10^ ^]^ Among angiosperms, several global studies have shown that diversification rates are highest in the species‐poor temperate regions^[^ ^4^, ^11^, ^12^ ^],^ paralleling the patterns seen in mountain birds. Together, these studies show that current diversification, i.e., over approximately the last 20 million years, is not always highest where species richness is highest, suggesting that species richness patterns are the result of deep evolutionary diversification rates^[^ ^13^ ^]^ and that they will shift over time, as species accumulate differently in regions with different diversity levels.

Ferns are the second largest group of vascular plants and occur almost worldwide with ≈12 000 extant species. Their species richness is highest at mid‐elevations in tropical mountains^[^ ^14^, ^15^, ^16^ ^],^ where phylogenetic endemism is also highest.^[^ ^17^ ^]^ In contrast, phylogenetic diversity, which reflects the full evolutionary history, tends to be highest at low elevations in ferns.^[^ ^18^ ^]^ Previous studies of phylogenetic diversity have commonly found that temperature‐related climatic factors are better predictors than precipitation‐related ones, and that climatic extremes play a greater role than climatic seasonality.^[^ ^17^ ^]^ Interestingly, phylogenetically old and isolated fern lineages that originated and diversified prior to the rise of angiosperms to ecological dominance in the Mesozoic often show different patterns than the modern polypod radiation, which evolved alongside the angiosperms.^[^ ^17^, ^19^ ^]^ However, tip diversification rates of ferns have not yet been explored, and it is unknown whether areas with high fern species richness have high or low diversification rates, nor whether the geographic distribution of diversification rates is related to climatic conditions.

Quantifying tip diversification rates can be done by several methodological approaches.^[^ ^20^ ^]^ One commonly used approach is the method‐of‐moments‐estimator,^[^ ^21^ ^]^ which is called the MS approach.^[^ ^22^ ^]^ This approach follows from the idea that a lineage's net diversification rate can be easily calculated by dividing the number of extant species in a lineage by its age.^[^ ^21^, ^22^, ^23^ ^]^ The MS approach is particularly useful because it does not require a detailed phylogeny for each lineage.^[^ ^22^ ^]^ Accordingly, this approach has been used to address many questions related to diversification in different groups of organisms, including angiosperms,^[^ ^21^, ^24^, ^25^, ^26^, ^27^ ^]^ bryophytes,^[^ ^10^, ^28^ ^]^ birds,^[^ ^29^, ^30^ ^]^ amphibians,^[^ ^31^, ^32^, ^33^, ^34^ ^]^ and fishes,^[^ ^35^, ^36^ ^]^ in addition to those covering multiple phyla of animals^[^ ^37^, ^38^ ^]^ and those covering both plants and animals.^[^ ^39^ ^]^ A relatively recently developed alternative approach, which is called Bayesian analysis of macroevolutionary mixtures (BAMM)^[^ ^40^ ^],^ has also been used in multiple studies. However, the reliability of the BAMM approach has been questioned.^[^ ^11^, ^41^ ^]^ Meyer & Wiens^[^ ^22^ ^]^ and Meyer et al.^[^ ^42^ ^]^ point out that BAMM gives misleading diversification rate estimates and that MS yields better results than BAMM.

In the present study, we use the MS approach to broadly explore tip diversification rates in ferns, specifically addressing the following topics. 1) We test whether geographic areas with warmer and wetter climates have fern species that belong to clades with higher diversification rates. 2) We explore variation in mean diversification rates of ferns across climatic gradients and among biomes. 3) We assess whether current climate variables have stronger effects on mean diversification rates than historical climate change variables, whether precipitation‐related climatic variables have stronger effects on mean diversification rates than temperature‐related climatic variables, and whether climate extreme variables have stronger effects on mean diversification rates than climate seasonality variables. 4) We test whether areas with higher species richness have ferns that belong to clades with higher diversification rates.

## 2 Results

### 2.1 Phylogenetic Signal of Diversification Rate

**Figure 1:**
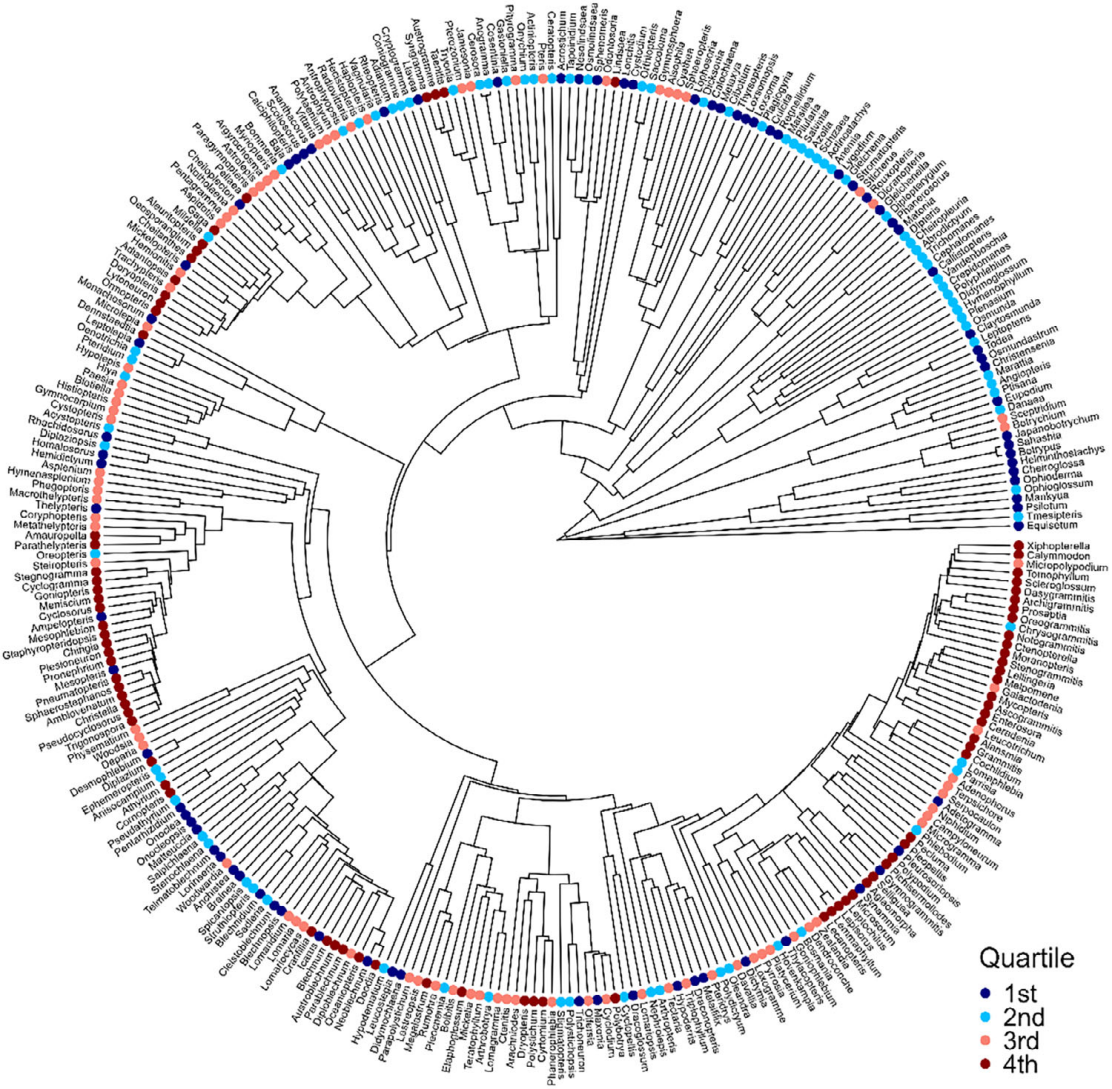
Diversification rates of fern genera across the phylogeny. For the purpose of displaying patterns of diversification rates, genera were divided into four quartiles according to diversification rate (from 1st to 4th quartile, corresponding to low to high diversification rate), each of which represented 25% of the genera. This phylogeny was extracted from the phylogeny of Nitta et al.^[^ ^63^ ^]^

Pagel's λ of diversification rates of ferns was 0.413 (*p* < 0.001). This indicated that closely related genera tended to have similar rates of diversification. This tendency can be clearly seen in **Figure** **1**. For example, the vast majority of the genera located in the upper‐right quarter of the phylogenetic tree, which were with dots in two cold colors, had low diversification rates and belonged to ancient lineages in the orders Equisetales, Psilotales, Ophioglossales, Marattiales, Osmundales, Hymenophyllales, Gleicheniales, Schizaeales, Salviniales, and Cyatheales. In contrast, the vast majority of the genera located in the lower‐right quarter of the phylogenetic tree, which were with dots in two warm colors, had high diversification rates and belonged to a single, relatively young family (i.e., Polypodiaceae).

### 2.2 Geographic Patterns of Mean Diversification Rate

**Figure 2:**
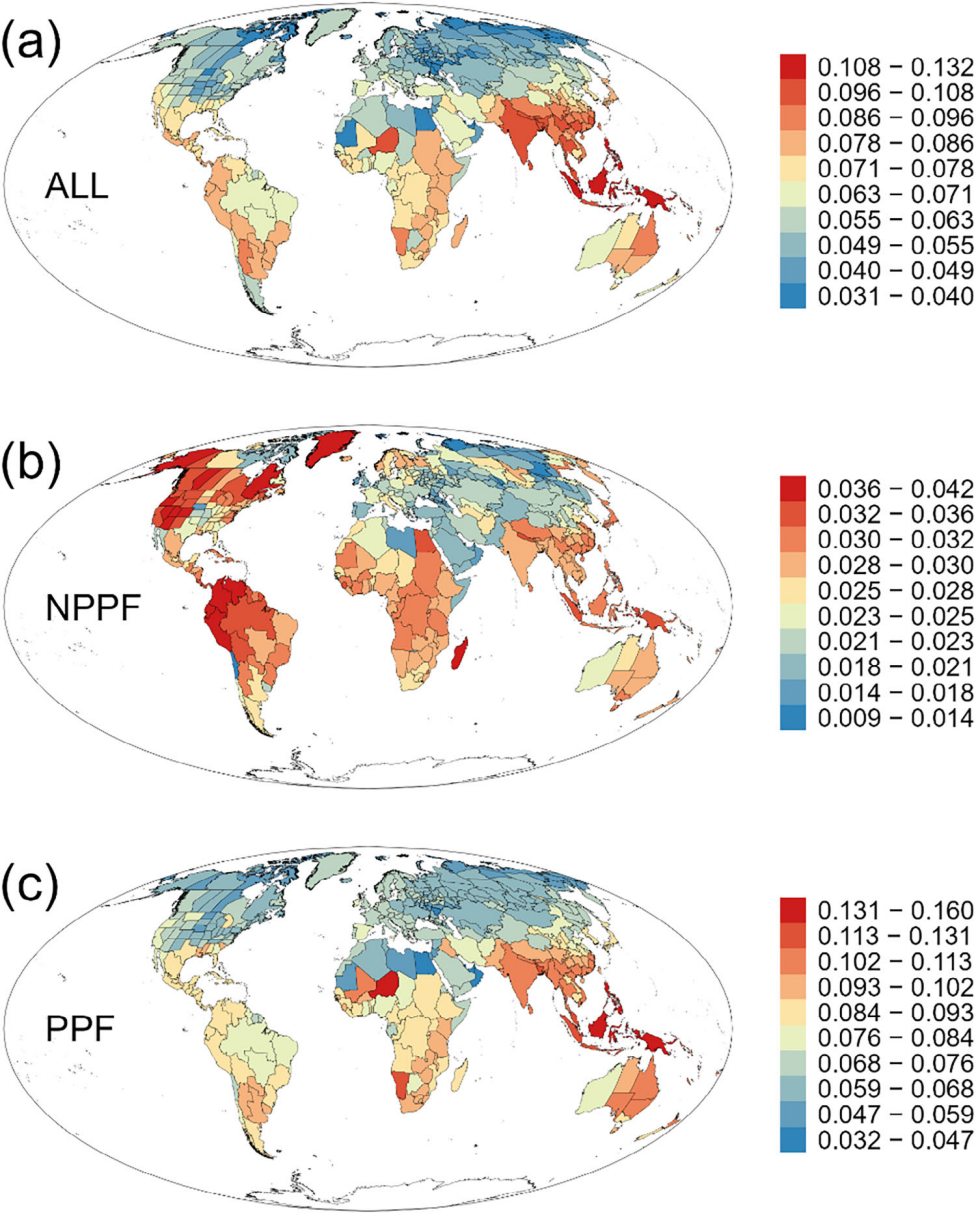
Geographic pattern of mean diversification rate (MDR) of all ferns (ALL), non‐polypod ferns (NPPF), and polypod ferns (PPF).

MDR varied greatly across the world and decreased with increasing latitude, regardless of whether all ferns were considered as a whole or polypod and non‐polypod ferns were considered separately (**Figure** **2** and **Table** **1**). For all ferns as a whole and polypod ferns, regions with the highest MDR were located in the Old World; however, for non‐polypod ferns, regions with the highest MDR were located in the New World (Figure 2).

**Table 1:** Spearman's rank correlations of mean diversification rate with latitude and variables representing current climate and Quaternary climate change for ferns in geographic units across the world.

Variable	All	Non‐polypod	Polypod
LAT	−0.751	−0.449	−0.680
T_mean_	0.643	0.354	0.604
T_min_	0.644	0.397	0.623
T_seas_	−0.668	−0.450	−0.655
P_mean_	0.535	0.485	0.526
P_min_	0.046	0.249	0.073
P_seas_	0.310	0.034	0.249
T_anom_	−0.550	−0.255	−0.516
P_anom_	−0.225	0.061	−0.180

Abbreviations of variables: LAT = absolute latitude, T_mean_ = mean annual temperature, T_min_ = minimum temperature of the coldest month, T_seas_ = temperature seasonality, P_mean_ = annual precipitation, P_min_ = precipitation during the driest month, P_seas_ = precipitation seasonality, T_anom_ = temperature anomaly, P_anom_ = precipitation anomaly.

**Figure 3:**
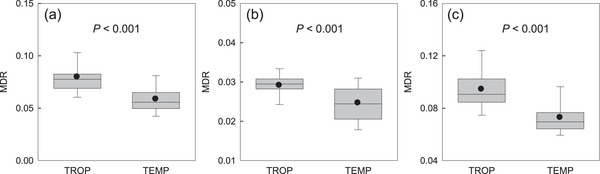
Comparison of mean diversification rate (MDR) of fern genera in each geographic unit between tropical (TROP) and temperate (TEMP) latitudes (a, all ferns; b, non‐polypod ferns; c, polypod ferns). The dots represent the mean, boxes the median and 25th and 75th percentiles, and whiskers the 10th and 90th percentiles. *P*‐values resulted from *t*‐test of the mean values of MDR between tropical and temperate geographic units.

MDR was significantly (*p* < 0.01) higher in tropical than in temperate zones (**Figure** **3**). This held true regardless of whether all ferns were considered as a whole or polypod and non‐polypod ferns were considered separately (Figure 3).

### 2.3 Relationships Between Mean Diversification Rate and Climatic Variables

**Figure 4:**
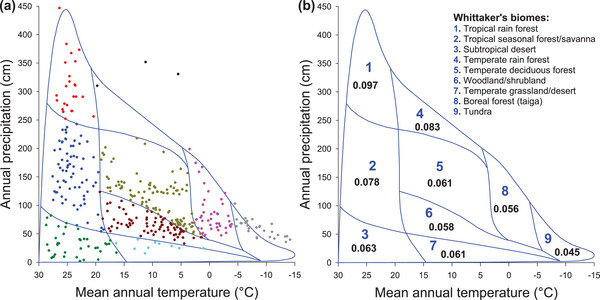
Distribution of geographic regions among the nine Whittaker's biomes (**a**) and the mean diversification rate of ferns for each of the biomes (**b**). Dots in the same colors belong to the same biomes.

When all genera of ferns were considered as a whole, MDR increased with mean annual temperature and annual precipitation (Spearman's correlation coefficient ρ = 0.643 and 0.535, respectively, Table 1). Three clear gradients of MDR across biomes emerged (**Figure** **4**). First, MDR decreased along the gradient from tropical rain forest (biome 1) → temperate rain forest (biome 4) → boreal forest (biome 8) → tundra (biome 9), corresponding to the decreasing gradient of MDR being 0.097 → 0.083 → 0.056 → 0.045 (Figure 4). Second, within warm climate conditions, MDR decreased along the gradient from tropical rain forest (biome 1) → tropical seasonal forest/savanna (biome 2) → subtropical desert (biome 3), corresponding to the decreasing gradient of MDR being 0.097 → 0.078 → 0.063 (Figure 4). Third, within dry climate conditions, MDR decreased along the gradient from subtropical desert (biome 3) → temperate grassland/desert (biome 7) → tundra (biome 9), corresponding to the decreasing gradient of MDR being 0.063 → 0.061 → 0.045 (Figure 4). Thus, among the nine biomes, tropical rain forest (biome 1) had the highest MDR, whereas tundra had the lowest MDR.

**Figure 5:**
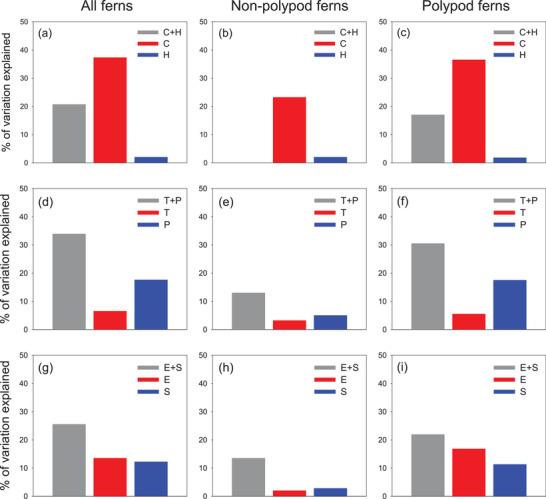
Variation in the mean diversification rate (MDR) of fern assemblages across the world is explained by different sets of climatic variables. a–c) Variation in MDR was explained jointly by current climate variables and historical climate change variables (C+H), independently by current climate variables (C), and independently by historical climate change variables (H). d–f) Variation in MDR was explained jointly by temperature‐related and precipitation‐related variables (T+P), independently by temperature‐related variables (T), and independently by precipitation‐related variables (P). g–i) Variation in MDR was explained jointly by climate extremes and seasonality variables (E+S), independently by climate extreme variables (E), and independently by climate seasonality variables (S). The value of C+H in b, which was negative, was not shown.

Current climatic variables played much larger roles in driving MDR than historical (Quaternary) climate change variables, regardless of whether all ferns were considered as a whole or polypod and non‐polypod ferns were considered separately (**Figure** **5**a–c). The amount of variation in MDR independently explained by current climatic variables was over 10 times larger than that independently explained by historical (Quaternary) climate change variables (Figure 5a–c). Among the current climatic variables, the three precipitation‐related variables explained more variation in MDR than did the three temperature‐related variables, regardless of whether all ferns were considered as a whole or polypod and non‐polypod ferns were considered separately (Figure 5d–f). The two climate extreme variables explained more variation in MDR when all ferns and polypod ferns were considered, but slightly less variation in MDR when non‐polypod ferns were considered, compared to the two climate seasonality variables (Figure 5g–i). When fern floras in the three longitudinal segments were analyzed separately (Figures S2–S4, Supporting Information), current climatic variables played much larger roles in driving MDR than historical climate change variables in all the longitudinal segments; temperature‐related variables explained more variation in MDR than precipitation‐related variables did in all the longitudinal segments for all ferns as well as for the two fern groups except for non‐polypod ferns in the western Old World (Figure S4, Supporting Information). The relative importance of climate extremes vs seasonality variables varied among the longitudinal segments (Figures S2–S4, Supporting Information).

**Figure 6:**
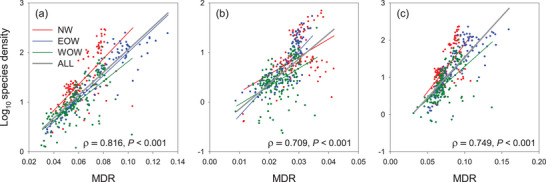
Relationships between mean diversification rate (MDR) and species density (species richness divided by log_10_‐transformed area) in geographic units for all ferns (a), non‐polypod ferns (b), and polypod ferns (c). Red dots and lines are for geographic regions in the New World (NW), blue dots and lines are for geographic regions in the eastern Old World (EOW), green dots and lines are for geographic regions in the western Old World (WOW), and thick grey lines are for all geographic regions combined (ALL). Each line is the best fit of linear regression to the data. Spearman's correlation coefficient (ρ) is for all geographic regions combined.

**Figure 7:**
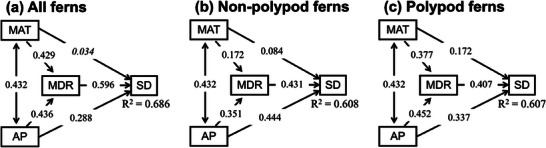
Each structural equation model depicts direct and indirect drivers of species density for all ferns (a), non‐polypod ferns (b), and polypod ferns (c). SD was log_10_‐transformed species density. Explanatory variables included mean annual temperature (MAT) and annual precipitation (AP), and mean diversification rate (MDR). All effects were significant (P < 0.05), except for the one in italic form (i.e., the path from MAT to SD in a).

MDR was positively correlated with species richness (Figure S1, Supporting Information) and species density (**Figure** **6**), with Spearman's correlation coefficient (ρ) ranging from 0.709 to 0.816. Our SEM analyses for fern floras across the world revealed several important results. First, precipitation had a stronger effect on species density than temperature, which was consistent with the aforementioned results derived from correlation analyses and variation portioning analyses for MDR (**Figure** **7**). Second, temperature and precipitation had stronger effects on MDR than on species density, except for the paths associated with precipitation in non‐polypod ferns (Figure 7). Third and most importantly, our SEM analyses showed that MDR had a positive and significant effect on species density (i.e., area‐corrected species richness) after accounting for the effects of temperature and precipitation on both MDR and species density (Figure 7). These results generally held true when fern floras in each of the three longitudinal segments were analyzed separately (Figure S5, Supporting Information).

## 3 Discussion

In this first study exploring the global variation of tip diversification rates in ferns, measured as mean diversification rate (MDR), we obtained the following main results. First, MDR peaked at tropical latitudes and in humid and hot environments, with MDR being influenced primarily by current climate (rather than historical climate change), by precipitation‐related variables (rather than temperature‐related ones), and roughly equally by climatic extremes and seasonality. Second, we found a positive relationship between MDR and fern species density, with the latter being more strongly directly influenced by MDR than by climate. Third, the above‐described patterns differ among longitudinal segments but less so between phylogenetically old fern lineages and the modern polypod lineage.

Three previous studies have shown that tip diversification rates of angiosperms are not highest in the tropics but rather in temperate regions.^[^ ^4^, ^11^, ^12^ ^]^ Our study for ferns shows the opposite pattern. Unfortunately, a direct comparison of the studies is not possible because of the different methods employed. Tietje et al.^[^ ^12^ ^]^ and Dimitrov et al.^[^ ^4^ ^]^ used Mean Root Distance (MRD) and BAMM, respectively, which both rely on information of branch splitting across a phylogeny from its root to its tips, potentially leading to biases.^[^ ^22^, ^41^, ^42^ ^]^ In contrast, the calculation of MDR as used by us does not take into account the relationships between genera and has been recommended for studies such as ours.^[^ ^22^, ^41^, ^42^ ^]^ However, in an unpublished pilot study using BAMM (M. Kessler, P. Lorenz et al. unpubl. data), the authors recovered similar patterns of fern diversification as found here, suggesting that the patterns reported here are methodologically robust.

Fern species richness and phylogenetic diversity are well known to peak at tropical latitudes, especially in humid mountain forests.^[^ ^14^, ^15^, ^16^, ^19^, ^43^ ^]^ We here find that these regions also have high values of MDR. It is crucial to bear in mind that our calculation is based on the mean values of MDR for each genus globally, so that a region with high MDR has to be interpreted as being populated by rapidly diversifying lineages, but this does not directly measure speciation events in that specific region. Nevertheless, the positive relationship between MDR and species density suggests that high MDR is a prerequisite for and likely contributor to high current speciation. The positive species‐richness−MDR relationship is unexpected for two reasons. First, our results contradict those found in angiosperms. Tietje et al.^[^ ^12^ ^]^ found that diversification rate and species richness are unrelated in angiosperms, while Dimitrov et al.^[^ ^4^ ^]^ found a negative relationship. In liverworts, MDR is also not linearly linked to species richness, being highest in tropical lowland forests where species richness is moderately high, and only intermediate at mid‐elevations in tropical mountains, where liverwort richness peaks.^[^ ^10^ ^]^ However, for mosses, a recent study^[^ ^28^ ^]^ found that MDR is positively correlated with species richness. These contrasting patterns suggest that the relationships between tip diversification rates and species richness might differ between major groups of land plants, reflecting their different evolutionary dynamics. Unlike angiosperms and liverworts, fern diversification appears to exhibit stronger niche conservatism due to their lower ability to control water loss and reliance on water‐assisted sperm transfer. This likely explains their contrasting responses to Cenozoic climate shifts, where ferns retained ancestral niche preferences, while angiosperms diversified via rapid adaptation. The lower diversification of liverworts may reflect their susceptibility to desiccation, constraining establishment in seasonal climates, although effective long‐distance spore dispersal in some clades^[^ ^17^ ^]^ may explain exceptions to regional niche conservatism. However, a direct comparison in diversification rate among different groups of organisms is difficult to make due to different methodological approaches and spatial scales used in different studies. For example, Tietje et al.^[^ ^12^ ^]^ used the BAMM approach to analyze data at a regional scale, whereas Maul et al.^[^ ^10^ ^]^ used the MS approach to analyze their data at local scales along elevational transects.

Second, and probably more importantly, there is evidence from various spatial and temporal scales that fern species richness might be saturated, i.e., is limited by interspecific competition. Weigand et al.^[^ ^15^ ^]^ found that local fern species richness (measured in plots of 400 m^2^) does not increase continuously with increasing regional species richness (measured in grid cells of 7666 km^2^) but levels off at high regional richness, suggesting that local fern richness may reach an upper bound independently of regional richness. This is supported by observations in Ecuador showing that local species richness declines in plots with a high overall abundance of ferns and dominance of a few highly competitive species.^[^ ^44^ ^]^ Likewise, on islands of different sizes in the Sundaic archipelago, Karger et al.^[^ ^45^ ^]^ observed that fern species have broader realized ecological niches on small islands with few species than on large, species‐rich islands, revealing an effect of interspecific competition. Of course, these studies only concern local scales, where interspecific interactions tend to be most pronounced^[^ ^46^ ^]^ and are not directly transferable to the regional scale studied here. However, using a global approach, Lehtonen et al.^[^ ^47^ ^]^ found that over evolutionary time scales, fern diversification rates show negative density dependence, declining in periods with a high diversity of genera, suggesting that there is an upper boundary to the richness of ferns that has repeatedly been approached in geological time. Our study suggests that this is not currently the case, and that species richness is increasing most strongly in regions that are already species‐rich. In angiosperms, the high diversification rates in temperate regions have been interpreted as showing that these regions have abundant empty niche space, favoring speciation^[^ ^4^, ^11^, ^12^ ^]^, but this does not appear to apply to ferns.

Our structural equation models show that the relationship between MDR and species richness in ferns is not simply a result of spurious covariation due to a direct influence of climate on both MDR and species richness. Rather, we find that the direct influence of MDR on fern richness is considerably higher than the direct influences of both temperature and precipitation, both of which have previously been shown to be strongly related to fern species richness and phylogenetic diversity.^[^ ^15^, ^16^, ^43^, ^48^ ^]^ This has been interpreted as reflecting physiological limitations of ferns to deal with water stress.^[^ ^15^, ^44^, ^48^ ^]^ Our study suggests that there is an additional component to this story, and that regions with high fern richness do not only have this high diversity because of suitable climatic conditions, but also because these regions are inhabited by genera with high diversification rates, although we must caution that high regional MDR does not directly quantify current speciation rates within the region. In any case, hybridization and polyploidization, both of which are common speciation modes among ferns, are likely to be more frequent where many species co‐occur^[^ ^49^, ^50^, ^51^ ^]^, providing a potential link between species richness and diversification rates.

We find that MDR is highest in tropical biomes with high temperatures and precipitation. These conditions are physiologically less stressful for plants and have thus been proposed to allow for high metabolic rates and fast generation cycles, which could drive high diversification rates.^[^ ^52^, ^53^ ^]^ Looking in more detail, our variation partitioning approach shows that while the combined effect of temperature and precipitation has the strongest correlation with MDR, the influence of precipitation alone is stronger than that of temperature alone. This result is in accordance with patterns found for species richness^[^ ^15^ ^]^ but contradicts those commonly found when relating climatic variables to phylogenetic diversity, where temperature typically has a stronger effect than precipitation.^[^ ^43^, ^48^ ^]^ Importantly, physiological stress on ferns is influenced both by precipitation and temperatures (frost is limiting at one extreme, high temperatures lead to high evapotranspiration at the other) and their interaction, so that a clear separation of their respective influences is challenging.^[^ ^43^, ^48^, ^54^ ^]^ Nevertheless, the different metrics of fern diversity and diversification are apparently differently influenced by climatic factors, although we are at present unable to fully explain these differences.

When further exploring the climatic variables, we find that the current climate has a consistently stronger influence on MDR than historical climate variability. This is unsurprising considering that tip diversification rates reflect the current evolutionary situation, but it is also in agreement with patterns seen for phylogenetic diversity.^[^ ^43^, ^48^ ^]^ We also found little difference between the influence of climatic extremes and seasonality on MDR, with a strong combined effect. Again, this differs from patterns in phylogenetic diversity, where climatic extremes consistently have a much stronger influence than climatic seasonality, both in ferns^[^ ^19^, ^43^ ^]^ and other plant groups.^[^ ^55^ ^]^ This has been interpreted as reflecting that the persistence of evolutionary lineages is more strongly limited by occasional extreme events, which are challenging for species to adapt to, than by climatic seasonality, which is more predictable and hence allows for easier physiological adaptation. The present study suggests that this might not apply to speciation. We suggest that this might be because newly evolving species tend to be physiologically very similar to each other, whereas different evolutionary lineages at a higher taxonomic level (genera, families, orders), whose diversity is reflected in metrics of phylogenetic diversity, often differ fundamentally in their physiology.

Previous studies on the evolutionary dynamics of ferns have found fundamental differences in patterns of species richness and phylogenetic diversity between phylogenetically old lineages that mostly originated prior to the rise of angiosperms to ecological dominance in the Mesozoic, and which often persist with few relictual genera^[^ ^47^ ^]^ and the modern polypod radiation, which tracked the angiosperm terrestrial revolution.^[^ ^56^, ^57^, ^58^ ^]^ However, in our study, we mostly only found minor differences between old (non‐polypod) lineages and polypods. Once again, this shows that MDR recovers a different evolutionary signal compared to metrics of phylogenetic diversity.

Interestingly, the above‐described global patterns do not always hold true when we separate the globe into three longitudinal segments, namely the New World, western Old World (Africa and Eurasia west of 75°E), and eastern Old World (Eurasia east of 75°E and Australasia). Thus, we find that the relative influence of climatic predictors shifts and that temperature emerges as a stronger predictor than precipitation in each longitudinal segment. Also, while climatic seasonality and extremes have similarly strong influences overall, in the eastern Old World, the sole influence of climatic extremes dominates, whereas the opposite is true in the western Old World. Comparing the current and historic climatic signals, the New World has a much stronger signal, whereas in the eastern Old World current climate is absolutely dominant. Perhaps most interestingly, when we relate MDR to species richness, we find that MDR is highest in East Asia from the Himalayas to New Guinea, whereas the fern floras of the tropical Andes, despite having higher species density, have somewhat lower values of MDR. Indeed, at similar levels of species richness, regional fern floras in the New World tend to have lower MDR than those in the eastern Old World. Interestingly, values of MDR in the fern floras of the western Old World do not differ markedly from those of the other continental regions, even though both Africa and Europe are well known for their low fern diversity.^[^ ^15^, ^16^, ^59^ ^]^ It is crucial in this context to remember that a low or high MDR in a particular region or environmental condition should not be taken to indicate that the region or environmental condition had a low or high diversification rate, but rather that the species assemblage of the region or environmental condition is composed of species from clades with low or high diversification rates. Bearing this in mind, we propose that Africa has unexpectedly high values of MDR because it has very few exclusive fern genera not shared with the other continents, so that our metrics, which calculate a global value for each genus, are unable to retrieve regional within‐genus variability, if any exists. These and other differences between continents reflect the combined effects of the different geographic features of the continental areas and of the evolutionary dynamics of their respective fern floras.^[^ ^43^, ^48^ ^]^

## 4 Conclusion

Our study shows that tip diversification rates tell a complementary story of the evolution of fern diversity to that recovered by studying phylogenetic diversity, which quantifies deeper evolutionary relationships. Most importantly, we found that regions of high fern diversity also have high current diversification rates, possibly due to positive species interactions such as hybridization and polyploidization, which might drive positive feedback between speciation rates and species richness. This results in the highest diversification in ferns in humid tropical regions, which contrasts with the observations of several studies in angiosperms^[^ ^4^, ^11^, ^12^ ^]^ and a study in liverworts^[^ ^10^ ^]^ where high diversification appears to occur mainly in regions with low to intermediate species richness and ample empty niche space. The different diversification dynamics between ferns and angiosperms might also be related to the highly efficient dispersal by spores in the former, which leads to stronger gene flow between populations. In angiosperms, allopatric speciation by geographic isolation is considered to be the dominant speciation mode,^[^ ^60^ ^]^ whereas in ferns, species interactions and adaptive processes might play a more important role, although this remains to be tested. Furthermore, ferns, angiosperms, and liverworts differ in their life histories, with ferns having two independent generations (gametophytic and sporophytic), whereas one generation dominates in angiosperms (sporophytic) and liverworts (gametophytic), which might also influence their respective diversification dynamics, e.g., through different generation times or differential selections on generations with different ploidy levels. Finally, it has been suggested that ferns diversify more rapidly in shady habitats that are common in tropical forests,^[^ ^61^ ^]^ possibly because their adaptations to low light conditions give them a competitive advantage over angiosperms.^[^ ^62^ ^]^ Such physiological differences between plant groups might explain why fern diversification dynamics may differ from those of other plant groups. Unfortunately, methodological differences between studies currently hamper a direct comparison, and a study of tip diversification rates across different plant groups using a consistent method and spatial scale would be desirable.

## 5 Experimental Section

### Estimation of the Diversification Rate of Ferns

The largest phylogeny of ferns to date includes less than half of the extant fern species;^[^ ^63^ ^]^ therefore, it is not robust to use a phylogeny‐based approach, such as BAMM,^[^ ^40^ ^]^ in a study investigating diversification rate at the species level for ferns. We used the MS approach based on the method‐of‐moments‐estimator^[^ ^21^, ^22^ ^]^ to calculate a lineage's net diversification rate as ln[*n*(1−ε) + ε]/*t*, where *n* is the number of extant species in the lineage, *t* is the age of the lineage, and ε is the relative extinction rate.^[^ ^21^, ^22^, ^23^ ^]^ Because ε is unknown, previous studies used values of ε ranging from 0 to 0.9.^[^ ^13^, ^21^ ^]^ Following previous studies (e.g.,^[^ ^13^ ^]^), three values of ε (i.e., 0, 0.5, 0.9) were used to estimate diversification rates for each genus. It was found that the three sets of diversification rates estimated using the three values of ε were nearly perfectly correlated with one another (Spearman's rank correlation coefficient ranging from 0.976 to 0.997), suggesting that using different values of ε would have little impact on the results of subsequent analyses on geographic patterns of diversification rates. Previous studies also show that using different values of ε has little impact on the results.^[^ ^13^ ^]^ Following previous authors (e.g.,^[^ ^10^, ^25^, ^27^ ^]^), the set of estimated diversification rates based on ε being set to zero in subsequent analyses were used. This setting was consistent with Louca & Pennell (2020), which demonstrated that net diversification rates remain identifiable even when extinction is poorly constrained. This conservative approach avoids overinterpretation of highly uncertain extinction estimates. Furthermore, this setting was appropriate because the current species richness in each lineage is the result of speciation minus extinction, which means that *n* in the above‐mentioned formula has already taken into account extinct species. This setting would be particularly appropriate for estimating recent diversification, such as diversification within genera, because diversification on tip branches should be less affected by extinction,^[^ ^11^, ^66^, ^67^ ^]^ compared with diversification on basal branches. Meyer & Wiens^[^ ^22^ ^]^ and Meyer et al.^[^ ^42^ ^]^ suggest that in the case of a lacking well‐resolved phylogeny for a lineage, the MS approach can be used to estimate the diversification rate of the lineage. Because tip rate metrics are more reliable estimators of speciation rate^[^ ^68^ ^]^ and because species within genera may be considered tip lineages and thus diversification rate within genera can be considered as tip rate of diversification, we applied the MS approach to calculate diversification rate for each fern genus, which is also called net diversification rate.^[^ ^10^ ^]^ Because of the challenges of estimating extinction, net diversification rates are often considered the most reliable metric.^[^ ^67^ ^]^

The phylogenies generated by Testo & Sundue,^[^ ^51^ ^]^ Hernández‐Rojas et al.,^[^ ^18^ ^]^ and Nitta et al.^[^ ^63^ ^]^ are the largest phylogenies for the global flora of ferns. Because each of these phylogenies has a unique strength, all of them were used to estimate genus ages. Fern names were standardized in the three phylogenies according to the database of World Ferns (https://www.worldplants.de/), using the R package U.Taxonstand.^[^ ^64^, ^65^ ^]^ A genus‐level phylogeny from each of the three mega‐phylogenies was extracted. These phylogenies included 90% of the genera of the extant global fern flora. For a given genus, stem age (i.e., the length of each tip branch) was extracted from each genus‐level phylogeny and calculated the average of genus stem ages from different phylogenies, which was considered as the age of the genus. Conflicting node ages across source phylogenies were resolved by calculating the mean stem age for each genus. Topological conflicts did not affect the analysis, as only stem ages (not internal relationships) were used to estimate diversification rates. This approach prioritizes age estimation robustness over topological congruence, as our method depends solely on stem age extraction. The number of species in each genus was extracted from the database of World Ferns (https://www.worldplants.de). The following formula was used to estimate the net diversification rate (*r*) of each genus:

(1) r=ln(n)/t

where *n* is the number of extant species in the genus and *t* is the stem age of the genus. For simplicity, it was called the diversification rate.

### Regional Fern Assemblages

Previous studies on global patterns in taxonomic and phylogenetic diversity of ferns have used 392 regional fern floras.^[^ ^17^, ^19^, ^43^ ^]^ The floras were compiled based on the data available in World Ferns (https://www.worldplants.de/) and Plants of the World Online (http://www.plantsoftheworldonline.org), which were supplemented with data from other sources. The same set of regional fern floras was used in this study. To explore variation in fern diversification rate among geographic regions across the world, the number of species in each genus in each of the 392 geographic regions was determined, which included 97% of extant fern species in the world. For each geographic region, each species was assigned the diversification rate of its genus. The mean diversification rate for each geographic region was the arithmetic mean diversification rate (MDR) across all species in the geographic region, as in Maul et al.^[^ ^10^ ^]^ It should be noted that a low or high MDR in a particular region or environmental condition should not be taken to indicate that the region or environmental condition had a low or high diversification rate; instead, a low or high MDR in a region or environmental condition reflects that the species assemblage of the region or environmental condition was composed of species from clades with low or high diversification rates, respectively.

Previous studies (e.g.,^[^ ^19^, ^69^ ^]^) have shown that geographic patterns of taxonomic and phylogenetic diversity differ substantially between polypod ferns (i.e., the order Polypodiales), which account for 82% of extant fern species, and other ferns (hereafter, non‐polypod ferns). Therefore, in addition to calculating MDR for ferns as a whole, MDR for polypod and non‐polypod ferns was also calculated separately.

The number of species (i.e., species richness) in each geographic region was determined, and followed the previous studies (e.g.,^[^ ^43^ ^]^) to determine area‐corrected species richness by dividing the number of species in each geographic region by the log_10_‐transformed area (in square kilometers) of the geographic region (hereafter, species density).

To explore variation in diversification rate between tropical and temperate latitudes, the globe was divided into two broad climate zones: the tropical zone (latitudes between 23.5° N and 23.5° S) and the temperate zone (latitudes south of 23.5° S or north of 23.5° N). A geographic region was assigned to either of the climatic zones according to the centroid of the latitude of the geographic region. Because previous studies on fern diversity^[^ ^15^, ^19^ ^]^ found marked differences between continental regions, the globe was further subdivided into three longitudinal segments, namely the New World, the western Old World (west of 75°E), and the eastern Old World (east of 75°E).

### Climate Data

The mean diversification rates of ferns in the 392 geographic regions were related to climatic variables reflecting current climatic conditions and historical climate change during the Quaternary, as previous studies have shown that they are drivers of geographic distributions of plants.^[^ ^43^, ^58^ ^]^ Specifically, current climatic variables included mean annual temperature (T_mean_), minimum temperature of the coldest month (T_min_), temperature seasonality (T_seas_), annual precipitation (P_mean_), precipitation during the driest month (P_min_), and precipitation seasonality (P_seas_); historical climate change variables included the differences in mean annual temperature and annual precipitation between the Last Glacial Maximum and the present, which are temperature anomaly (T_anom_) and precipitation anomaly (P_anom_), respectively. T_mean_, T_min,_ and T_seas_ were considered as temperature‐related variables of current climate and P_mean_, P_min,_ and P_seas_ as precipitation‐related variables; T_min_ and P_min_ were considered as climate extreme variables and T_seas_ and P_seas_ as climate seasonality variables in current climate conditions. Data for the climatic variables for each geographic region were obtained from Qian et al.,^[^ ^43^ ^]^ which were derived from data in the CHELSA climate database (https://chelsa‐climate.org/bioclim) and Sandel et al.^[^ ^70^ ^]^

### Data Analysis

Pagel's *λ* ^[^ ^71^ ^]^ was used to assess whether there is a significant phylogenetic signal in diversification rate, using the genus‐level phylogeny derived from Nitta et al.^[^ ^63^ ^]^ and the function phylosig in the package phytools.^[^ ^72^ ^]^ A value of 0 indicates no phylogenetic signal in diversification rate across the phylogeny, whereas a value of 1 indicates a strong phylogenetic signal, which matches expectations under the Brownian motion model.

A *t*‐test was conducted to assess differences in MDR in geographic regions between tropical and temperate zones. The relationship of MDR was assessed in geographic regions with latitude and each of the climatic variables using Spearman's rank correlation (ρ).

Temperature and precipitation are the most important drivers of plant distributions, and biomes are often defined based on these two climate axes (e.g.,^[^ ^73^ ^]^). To explore patterns of variation in MDR among biomes across the space of temperature and precipitation and among biomes, each of the 392 geographic regions was assigned to one of Whittaker's biomes. For each of a small number of the geographic regions that were located outside Whittaker's biome framework, we assigned it to its closest biome. The number of geographic regions assigned to each of Whittaker's biomes is as follows: tropical rain forest (25 geographic regions), tropical seasonal forest/savanna (70), subtropical desert (38), temperate rain forest (3), temperate deciduous forest (101), woodland/shrubland (73), temperate grassland/desert (10), boreal forest (taiga) (43), and tundra (29). The mean value of MDR was calculated for each biome.

To determine the effects of different groups of environmental variables independently and jointly on MDR, three sets of variation partitioning analyses was conducted^[^ ^74^ ^]^ to partition the explained variation into different portions based on the adjusted coefficient of determination. The first variation partitioning analysis determined the relative effects of current climatic variables and historical climate change variables on MDR, which partitioned the amount of the explained variation in MDR into three portions: variation explained uniquely by current climatic variables, variation explained uniquely by historical climate change variables, and variation explained jointly by the two sets of variables. The second variation partitioning analysis determined whether temperature‐related variables or precipitation‐related variables in current climates have a stronger influence on MDR. The third variation partitioning analysis determined whether climate extreme variables or climate seasonality variables have a stronger influence on MDR. The package SYSTAT v.10 was used^[^ ^75^ ^]^ for the above‐described statistical analyses.

A structural equation modeling (SEM) approach was used to examine the direct and indirect effects of mean annual temperature, annual precipitation, and mean diversification rate on species density. The framework of the SEM was built based on the assumptions that i) species richness is influenced by diversification and environmental variables, and ii) diversification is influenced by environmental variables. Here, we focused on two major environmental variables (i.e., temperature and precipitation) that have been broadly considered to influence species richness and diversification of plants. In the framework of the SEM, mean annual temperature and annual precipitation were exogenous variables, and the mean diversification rate was an endogenous variable. Using structural equation modeling, whether diversification rate influences species richness when the influences of major climatic factors on both diversification rate and species richness were accounted for was explicitly tested. The R package “lavaan” (https://cran.r‐project.org/web/packages/lavaan) was used for the SEM analyses.

### Data Accessibility Statement

Fern distribution data are available at the World Ferns database (https://www.worldplants.de) and the Plants of the World Online database (https://powo.science.kew.org). Climate data that were used in the present study are available at the CHELSA (https://chelsa‐climate.org/bioclim). Sources of other data used in this study were cited in the article.

## Conflict of Interest

The authors declare no conflict of interest.

## Author Contributions

H.Q. and M.K. contributed equally to this work. H.Q. developed the original idea of the analyses presented in the manuscript. H.Q. and M.K. wrote the original draft of the manuscript. S.Q. conducted analyses. All authors contributed significantly to improving the manuscript.

## Supporting information



Supporting Information

Supporting Information

## Data Availability

The data that support the findings of this study are openly available in World Ferns database at https://www.worldplants.de. These data were derived from the following resources available in the public domain: https://www.worldplants.de.
