# Supplementary material for: Global Diversification Rates of Ferns Across Spatial and Climatic Gradients
Source: Adv Sci (Weinh). 2025 Nov 5;13(3):e08106. doi: 10.1002/advs.202508106 (PMC12806278; doi:10.1002/advs.202508106)
Supplement: Supplementary file 1 — Supporting Information [file ADVS-13-e08106-s002.doc]

**Supporting Information**

**Global Diversification Rates of Ferns across Spatial and Climatic Gradients**

Hong Qian,^*^ Michael Kessler, and Shenhua Qian

Figure S1. Relationships between mean diversification rate (MDR) and species richness in geographic units for all ferns (a), non-polypod ferns (b), and polypod ferns (c). Each red line is the fit of linear regression model to the data, and the dash blue lines represent 95% confidence intervals.

Figure S2. Variation in mean diversification rate (MDR) of fern assemblages across the New World explained by different sets of climatic variables. (a−c) Variation in MDR was explained jointly by current climate variables and historical climate change variables (C+H), independently by current climate variables (C), and independently by historical climate change variables (H). (d−f) Variation in MDR was explained jointly by temperature-related and precipitation-related variables (T+P), independently by temperature-related variables (T), and independently by precipitation-related variables (P). (g−i) Variation in MDR was explained jointly by climate extreme and seasonality variables (E+S), independently by climate extreme variables (E), and independently by climate seasonality variables (S). Negative values were not shown.

Figure S3. Variation in mean diversification rate (MDR) of fern assemblages across the eastern Old World explained by different sets of climatic variables. (a−c) Variation in MDR was explained jointly by current climate variables and historical climate change variables (C+H), independently by current climate variables (C), and independently by historical climate change variables (H). (d−f) Variation in MDR was explained jointly by temperature-related and precipitation-related variables (T+P), independently by temperature-related variables (T), and independently by precipitation-related variables (P). (g−i) Variation in MDR was explained jointly by climate extreme and seasonality variables (E+S), independently by climate extreme variables (E), and independently by climate seasonality variables (S). Negative values were not shown.

Figure S4. Variation in mean diversification rate (MDR) of fern assemblages across the western Old World explained by different sets of climatic variables. (a−c) Variation in MDR was explained jointly by current climate variables and historical climate change variables (C+H), independently by current climate variables (C), and independently by historical climate change variables (H). (d−f) Variation in MDR was explained jointly by temperature-related and precipitation-related variables (T+P), independently by temperature-related variables (T), and independently by precipitation-related variables (P). (g−i) Variation in MDR was explained jointly by climate extreme and seasonality variables (E+S), independently by climate extreme variables (E), and independently by climate seasonality variables (S). Negative values were not shown.


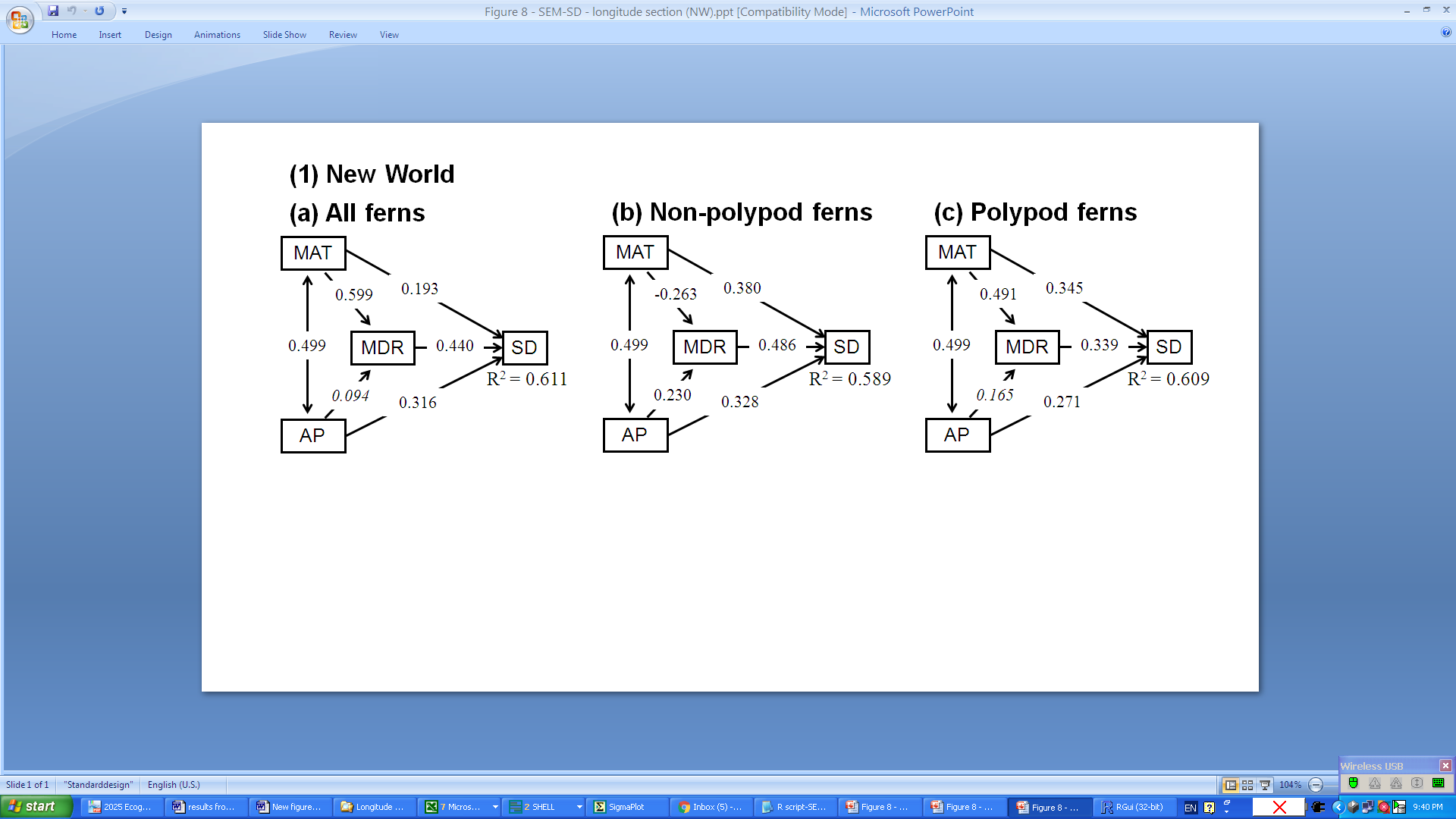


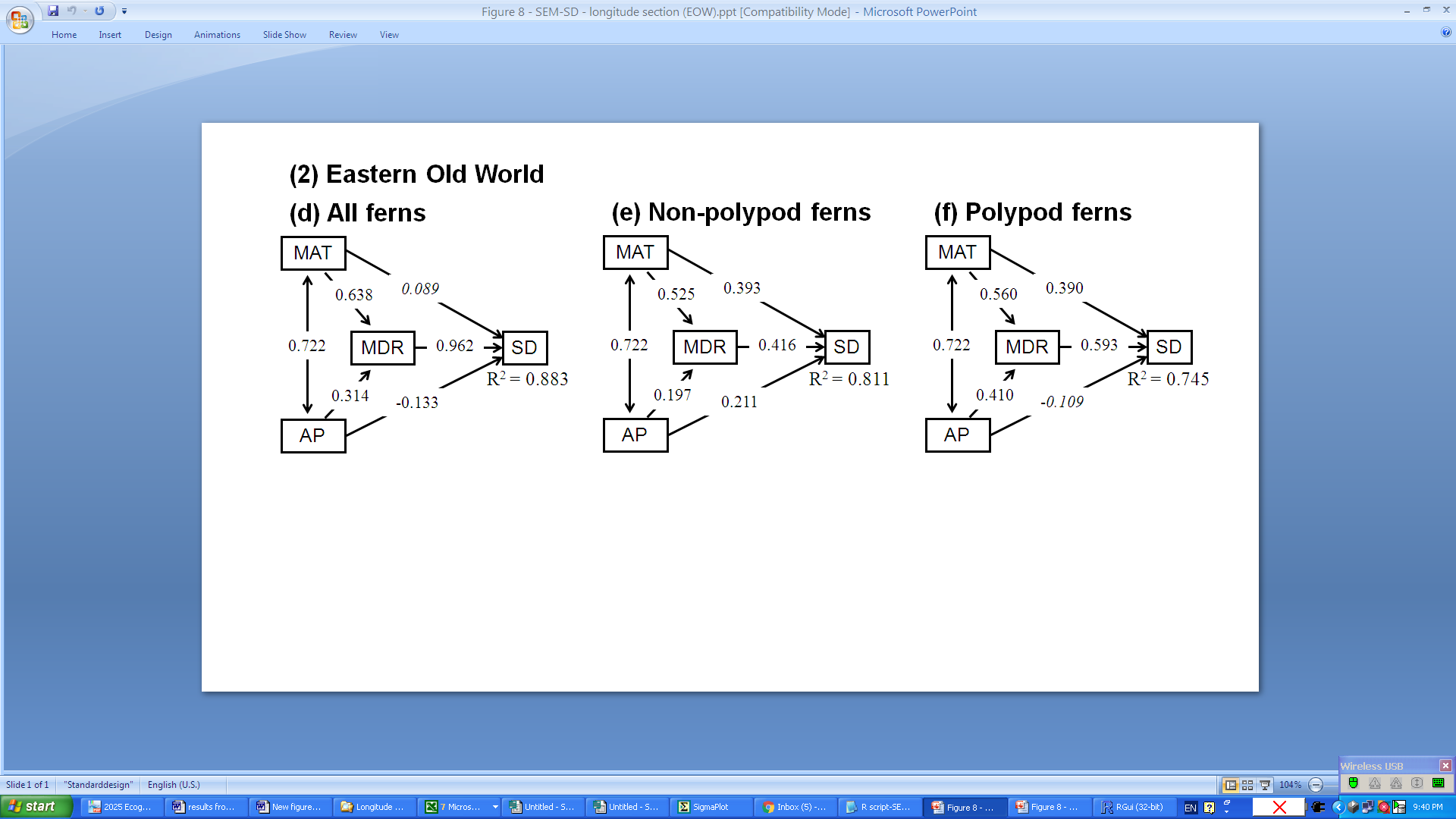


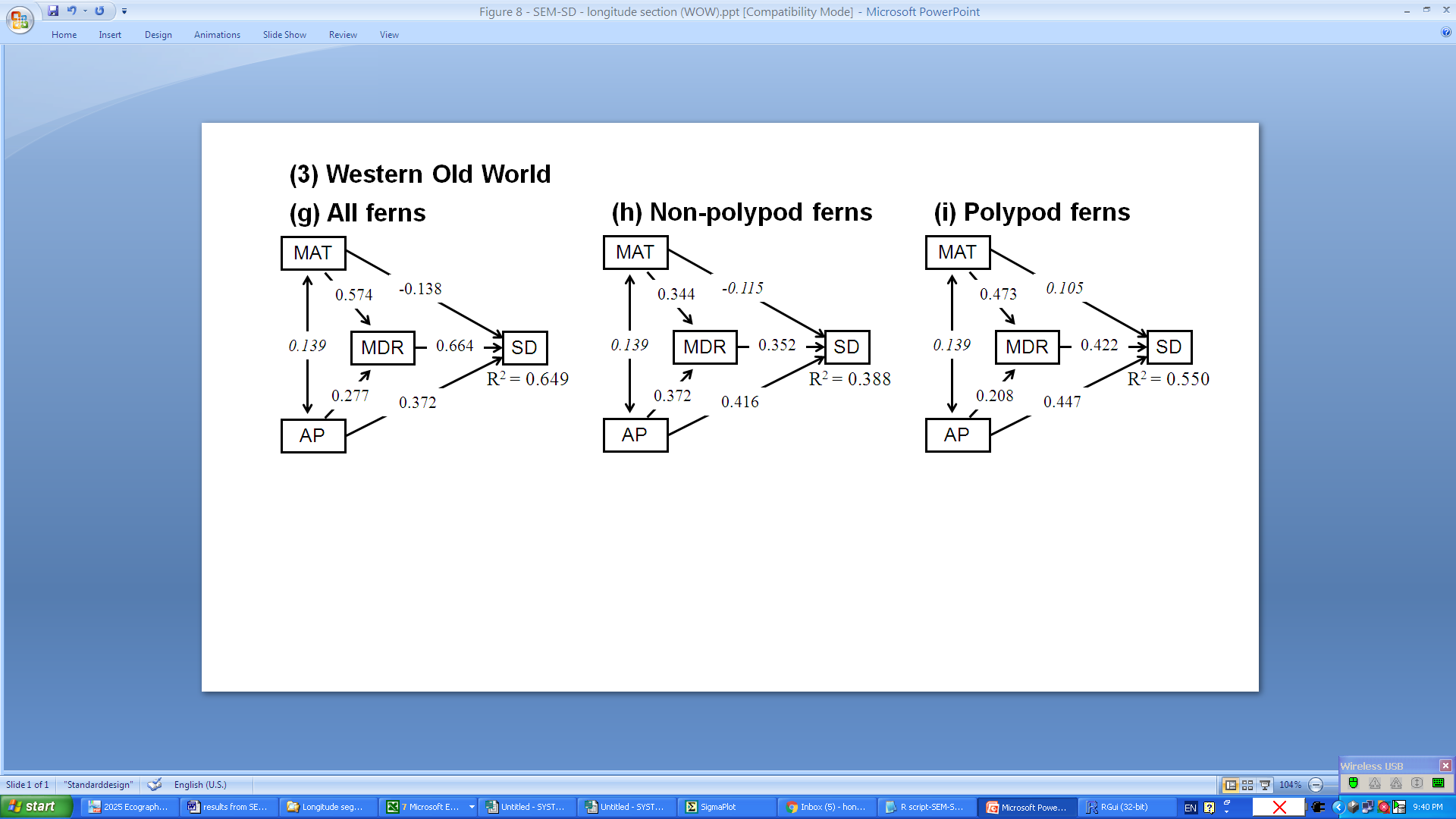


Figure S5. Each structural equation model depicts direct and indirect drivers of species density for all ferns, non-polypod ferns, and polypod ferns in the New World, the eastern Old World, and western Old World. SD was log_10_-transformed species density. Explanatory variables included mean annual temperature (MAT) and annual precipitation (AP), and mean diversification rate (MDR). All effects were significant (P < 0.05), except for those in italic form.
